# Combined evaluation of bone marrow aspirate and biopsy is superior in the prognosis of multiple myeloma

**DOI:** 10.1186/1746-1596-5-30

**Published:** 2010-05-18

**Authors:** Sanja Štifter, Emina Babarović, Toni Valković, Irena Seili-Bekafigo, Christophe Štemberger, Antica Načinović, Ksenija Lučin, Nives Jonjić

**Affiliations:** 1 Department of Pathology, School of Medicine, University of Rijeka, Braće Branchetta 20, 51000 Rijeka, Croatia; 2 Department of Hematology, University Department of Medicine, University Hospital Center Rijeka, Cambierieva 17, 51000 Rijeka, Croatia; 3 Department of Cytology, University Department of Medicine, University Hospital Center Rijeka, Cambierieva 17, 51000 Rijeka, Croatia

## Abstract

**Background:**

Estimation of plasma cell infiltrates in bone marrow aspirates (BMA) and bone marrow biopsy (BMB) is a standard method in the diagnosis and monitoring of multiple myeloma (MM). Plasma cell fraction in the bone marrow is therefore critical for the classification and optimal clinical management of patients with plasma cell dyscrasias. The aim of the study was to compare the percentage of plasma cells obtained by both methods with the patient clinical parameters and survival.

**Methods:**

This retrospective study included BMA and BMB of 59 MM patients. The conventional differential count was determined in BMA to estimate the percentage and cytologic grade of plasma cells. The pattern of neoplastic infiltration and percentage of plasma cells were estimated on CD138 immunostained BMB slides microscopically and by computer-assisted image analysis (CIA).

**Results:**

Significantly higher values of plasma cell infiltrates were observed in pathologist (47.7 ± 24.8) and CIA (44.1 ± 30.6) reports in comparison with cytologist analysis (30.6 ± 17.1; *P *< 0.001 and *P *< 0.0048, respectively). BMB assessment by pathologist counting and using CIA showed strongest correlation (r = 0.8; *P *< 0.0001). Correlation was also observed between the pathologist and cytologist counts (r = 0.321; *P *= 0.015) as well as comparing the percentage of plasma cells in BMA and CIA (r = 0.27; *P *= 0.05). Patients with clinical stage I/II had a significantly lower CIA plasma cell count than those with clinical stage III (*P *= 0.008). Overall survival was shorter in patients with more than 25% of atypical plasma cell morphology estimated in BMA (*P *= 0.05) and a higher percentage of tumor cell infiltrates estimated by the pathologist and CIA (*P *= 0.0341 and *P *= 0.013, respectively).

**Conclusion:**

Study results suggested the combined analyses to be useful as a routine procedure to achieve more accurate and informative diagnostic data.

## Introduction

Bone marrow analysis is an important element in establishing the diagnosis of multiple myeloma (MM), regardless of the indicative immunology or radiology findings [1,2]. It provides necessary information on the level of bone marrow involvement by plasma cells and its morphological specificities [2]. Minor and major criteria for the diagnosis according to the definition of the WHO classification include different categories of the bone marrow plasma cell count: a shift from 10%-30% group to >30% group equals shift from minor to major criteria, while <10% group does not contribute to the diagnosis [3]. In addition, plasma cell quantification is used in the evaluation of morphological remission [4] and minimal residual disease in MM patients [5]. A high percentage of plasma cells infiltration in bone marrow have been also recognized as a reliable predictor of relapse in cases of treated MM, as well as plasma cell microaggregates detected by immunohistology [6]. Plasma cell fraction in the bone marrow is therefore critical for the classification and optimal clinical management of patients with plasma cell dyscrasias.

Bone marrow aspirate (BMA) is essential for appropriate evaluation of plasma cell differentiation. On the basis of plasma cell morphology in BMA, myelomas can be classified into mature, intermediary, immature and plasmablastic [7]. It has been demonstrated that this division correlates with patient survival, since those with plasmablastic morphology have median survival of 10 months *versus *35 months for all other types among which no significant difference in survival was observed [7,8]. In comparison with BMA, trephine bone marrow biopsy (BMB) is not most suitable for the analysis of atypical plasma cells because of very difficult morphology identification of plasmablastic, lymphoid, lobated nucleus and polymorphic plasma cells.

On the other hand, plasma cell infiltrates in bone marrow with increased reticulum fibers that can be observed in nearly 9% of MM are preferably estimated in BMB rather than BMA because reticuloplasia often leads to scanty cellular aspirates. In addition, studies have shown BMB to enable plasma cell infiltrate classification into interstitial, nodular and diffuse types [9,10]. The amount of bone infiltrate varies from small clusters in otherwise normocellular bone marrow up to diffuse 100% bone marrow infiltration. The type of infiltration pattern is in proportion with the stage of disease. The interstitial and nodular patterns are observed when hematopoiesis is still preserved. In contrast, diffuse infiltration results in suppression of hematopoiesis. Transformation from interstitial or nodular towards diffuse infiltrate is observed as the disease progresses.

Since accurate quantification of bone marrow plasma cells is an important step in the diagnosis and post-treatment assessment of plasma cell dyscrasias, the aim of the present study was to contribute to the current view of the importance of evaluating both BMA and BMB. At the same time, the value of image analyses in the diagnostic work-up remains to be determined. Thus, another objective of the study was to compare the plasma cell percentage estimated in BMA and in CD138 stained BMB evaluated microscopically or by computer-assisted digital image analysis (CIA), and then to compare these values with the patient clinical parameters, therapy response and survival.

## Patients and Methods

This retrospective study included 59 patients diagnosed with MM at Department of Hematology, University Department of Medicine, Rijeka University Hospital Center, during the 2001-2008 period. The diagnosis of MM was established using the International Myeloma Working Group 2003 diagnostic criteria [11]. The patient clinical characteristics and clinical stage at the time of diagnosis according to Durie-Salmon staging system are presented in Table 1. The median age of patients at diagnosis was 71 (range 42-90) years. There were 33 female and 26 male patients (female to male ratio 1.3:1). The study was approved by the University of Rijeka Ethics Committee.

**Table 1:** Clinical characteristics of multiple myeloma (MM) patients

Characteristic		No. (%) of MM patients
Sex	Male	26 (44%)
	Female	33 (56%)
	M:F	1:1.3
Age (yrs)	Range	42-90
	Median	71
Clinical stage	I	8 (13.6%)
	II	23 (39.0%)
	III	28 (37.4%)

Total		59 (100%)

Treatment regimens were heterogeneous but in most cases included VAD (vincristine, doxorubicin and dexamethasone) infusion chemotherapy, melphalan and prednisolone, thalidomide alone or with dexamethasone, high-dose dexamethasone, and bortezomib alone or with dexamethasone. Some patients were treated with high-dose medicamentous therapy and autologous stem cell transplantation. All patients with bone disease were administered bisphosphonates and some of them also received local radiotherapy.

Therapeutic response was defined according to the EBMT, IBMTR/ABMTR criteria [12]; however, due to the small number of cases in some response groups, the patients were divided into two groups: those without any response or with refractory/progressive disease and those that achieved some response (complete, partial or minimal) to therapy. The follow up period was minimally 24 months (24-106 months).

Analysis of BMA included determination of plasma cell percentage from 400-cell differential count on conventional May-Grünwald-Giemsa stained aspirates, and quantification of atypical plasma cells classified according to cytologic grading of neoplastic cells as mature and those expressing signs of atypia (in differentiation) according to the WHO criteria.

Biopsy specimens (BMB) were fixed in Schaffer fixative for 24 hours and decalcified in Osteodec over 4-5 hours (Merck). Routine hemalaun-eosin (HE) staining, Giemsa, Periodic Acid Schiff (PAS), Gomory and Prussian blue for Fe detection were used. The presence of marrow fibrosis and normal hematopoiesis were also recorded.

Slides with 4-μm thick sections were deparaffinized in xylene, hydrated in graded alcohols and immunohistochemically stained with anti-CD138, Ig kappa and Ig lambda antibodies. Histology analysis included assessment of bone marrow infiltrate (diffuse, nodular, interstitial), percentage of tumor plasma cells in bone marrow specimen (CD138) and restriction of Ig light chains, quantifying the overall marrow cellularity as percentage of myeloma cells in biopsy specimen.

All slides stained with anti-CD138 were scanned and analyzed with Alphelys Spot Browser 2 integrated system, using a software controlled (Alphelys Spot Browser 2.4.4., France) stage positioning Nikon Eclipse 50i microscope mounted 1360 × 1024 resolution Microvision CFW-1310C digital camera. Overview images were taken at ×20 magnification and analysis images at ×100 magnification.

### Statistical analysis

The difference in average (median) number of plasma cell counts was tested with exact Mann-Whitney test. The correlation between the values recorded was assessed with exact Pearson/Spearman rank coefficient. The probability values of *P *< 0.05 were considered statistically significant. The Kaplan-Meier method was used to estimate overall patient survival. The SPSS version 12.0 software (SPSS, Chicago, IL, USA) was used on all analyses.

## Results

### Bone marrow plasma cell infiltrates assessed in BMA and BMB

**Figure 1:**
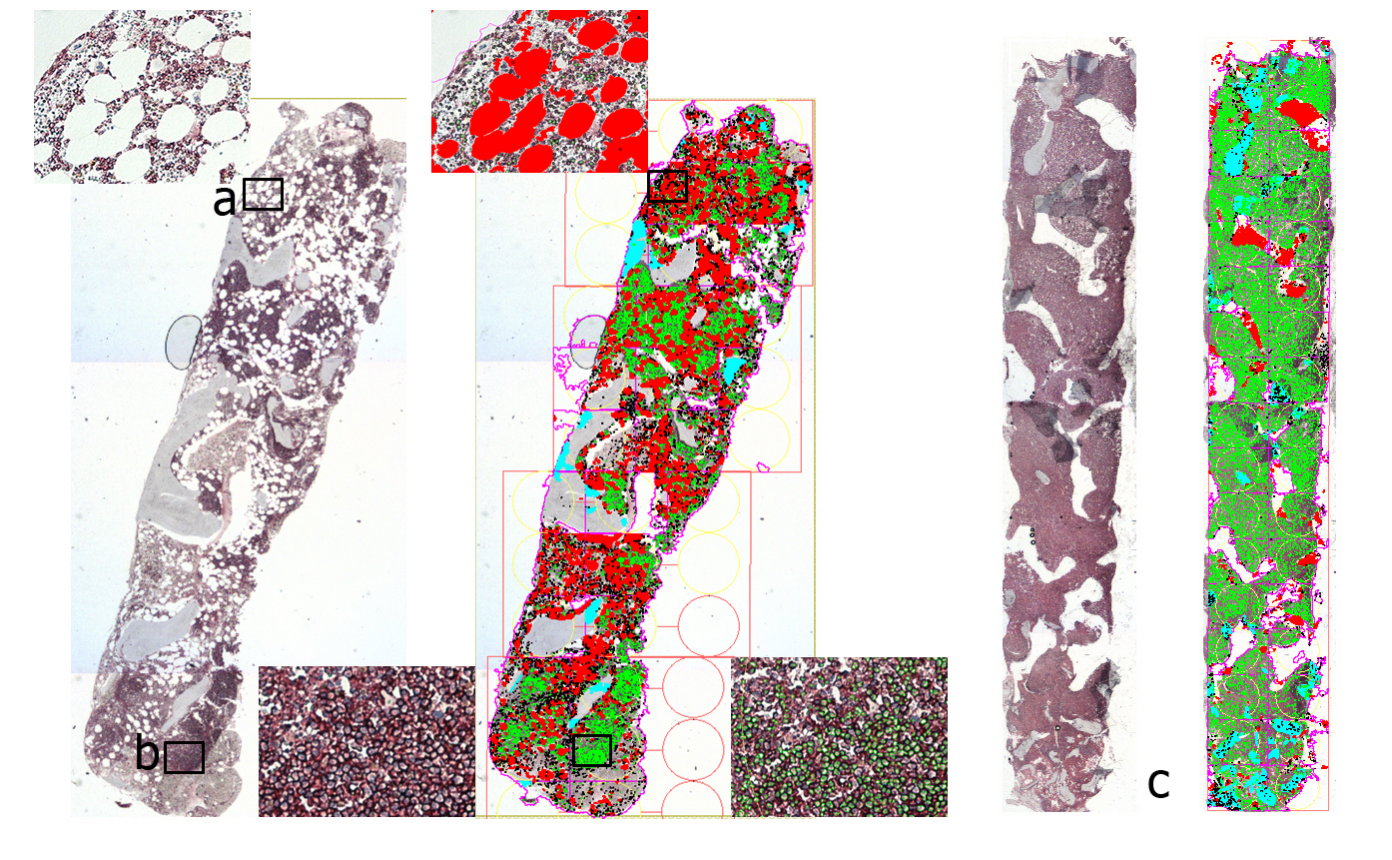
**Computer-assisted image analysis**. Screen shoots of scanned sample, with detailed histology images of (a) interstitial, (b) nodular and (c) diffuse type of bone marrow infiltration showing color based sample analysis.

In general, CD138 stained BMB consistently demonstrated greater plasma cell infiltration as compared with BMA. On image analysis, software detected cells and bone marrow areas based on their color properties (wave length, intensity and saturation) and morphometry (size and shape). The software was programmed to detect section area from background empty space, white areas representing clear areas of fat cells, homogeneous blue areas representing bone marrow trabeculae, heterogeneous cellular marrow area, 'positive' brown cells and 'negative' blue cells. Counting of CD138 positive and negative cells was only performed in regions of cellular marrow. Plasma cell percentage was calculated for each section based on its positive and negative cell count. Section area, bone marrow trabeculae area, fat cell area and cellular marrow area were calculated automatically by the software (Figure 1).

**Figure 2:**
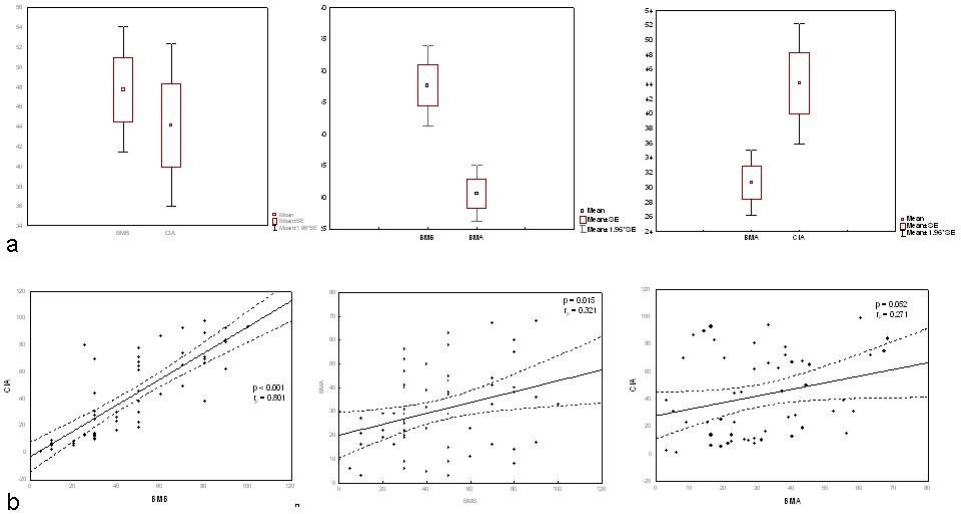
**Analysis of plasma cell count in bone marrow aspirate (BMA) and bone marrow biopsy (BMB)**. (a) Box and whisker plot (T-test) showing significant difference in plasma cell count between BMA and BMB analysis. Higher values were observed in pathologist (BMB) (mean 47.712 ± 24.8) and computer image analysis (CIA) (mean 44.113 ± 30.6) as compared with (BMA) cytologist report (mean 30.649 ± 17.1) (*P *< 0.001 and *P *< 0.0048, respectively). (b) Scatter plots showing correlation between CIA and BMB; BMA and BMB; CIA and BMA. Correlation of BMB assessment: pathologist and CIA counting (r = 0.8; P < 0.0001), pathologist and cytologist counting (r = 0.321; *P *= 0.015), and percentage of plasma cells in BMA and CIA (r = 0.27; *P *= 0.05).

The median percentage of plasma cell infiltrate recorded in BMA was 29% (range 3%-68%) and in BMB 50% (range 5%-100%) when assessed by the pathologist and 39% (range 1%-99%) with the use of CIA. Statistical analysis with T-test for independent samples showed significant difference between BMA and BMB, whereas no difference was observed between the pathologist and CIA counts (*P *= 0.49). More precisely, significantly higher values were observed in the pathologist's (mean 47.712 ± 24.8) and CIA reports (mean 44.113 ± 30.6) as compared with the cytologist's report (mean 30.649 ± 17.1; *P *< 0.001 and *P *< 0.0048, respectively) (Figure 2a). This discrepancy was mainly present in cases with nodular (8.5%) or nodular/interstitial (10.2%) pattern of bone marrow involvement and marked reticuloplasia.

Plasma cells with cytologic atypia on BMA were observed in 45.6% (26/58) of cases. Comparison by Pearson test yielded a correlation of plasma cell quantification between BMA and CD138 stained BMB sections reviewed microscopically by the pathologist and CIA. However, the two methods of BMB assessment, i.e. counting by the pathologist and by use of CIA, showed highest correlation (r = 0.8; *P *< 0.0001). Correlation was also recorded between plasma cell assessments done by the pathologist and cytologist (r = 0.321; *P *= 0.015), and between plasma cells in BMA and CIA (r = 0.27; *P *= 0.05) (Figure 2b).

### Comparison of plasma cell infiltrates with clinical parameters

**Figure 3:**
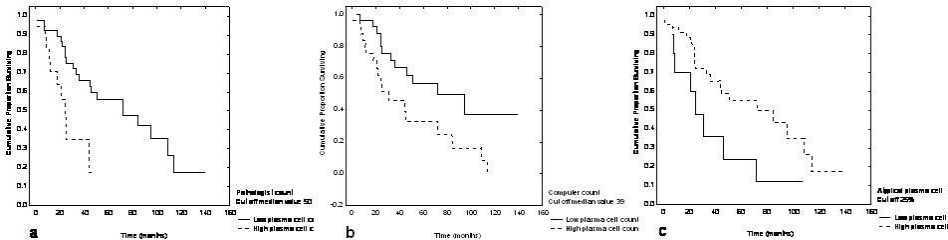
**Kaplan-Meier survival analysis (months) showing difference in patients survival (when plasma cell count divided in high and low groups according the median) obtained by (a) pathologist report (BMB) (*P *= 0.0341), (b) computer-assisted (CIA) report (*P *= 0.013), and (c) cytologist (BMA) report (*P *= 0.05), when cut off for atypical plasma cell count was set at 25%**.

For statistical analysis, patients were regrouped into clinical stage I/II *versus *stage III. Mann-Whitney test yielded significant difference for CIA plasma cell count (*P *= 0.008) (Table 2). Kaplan-Meier survival analysis (months) showed a mean survival time (limited to median) of 25 months. When patients were divided in two groups, with high and low plasma cell count, according to median percentage of plasma cell infiltrate, e.g. 50% in BMB, 39% in CIA, and 29% in BMA, the analysis showed difference in survival when plasma cell count reported by the pathologist (*P *= 0.0341) (Figure 3a) and CIA (*P *= 0.013) (Figure 3b), but not with the cytologist's report (*P *= 0.951). As the values of atypical plasma cell increased by more than 25% (of atypical plasma cells in whole plasma cell count), survival time significantly decreased (*P *= 0.05) (Figure 3c).

**Table 2:** Comparison of plasma cell count recorded in bone marrow biopsy (BMB), bone marrow aspirate (BMA) and by computer-assisted image analysis (CIA) according to clinical stage

Clinical stage			
	**I, II**	**III**	***P***

BMB	44.68 ± 23.5	51.01 ± 26.12	0.239
BMA	27.26 ± 16.0	34.67 ± 17.88	0.136
CIA	35.54 ± 28.0	54.06 ± 31.02	0.008

## Discussion

The quantification of plasma cells in bone marrow is an essential step in the diagnosis of plasma cell dyscrasias. At this point, one can argue that the acquired bone marrow particles dispersed in BMA are sufficient for microscopic diagnosis in most cases [5,12]. However, MM is a focal process, in our study in nearly 20% of cases, and it may have some impact on the accuracy and reliability of the plasma cell percentage assessment in BMA. As demonstrated elsewhere, there are notable discrepancies and a relatively poor correlation in determining the percentage of bone marrow plasma cells between BMA and BMB [13]. So, many studies have been conducted in order to recommend the most reliable method of estimating plasma cell infiltrates that could be routinely performed.

In the present study, an immunohistochemistry method with CD138 on BMB was used. The utility of plasma cell quantification by CD138 immunohistochemistry has been widely supported [6,14-16]. It has been generally accepted as the most sensitive method for quantifying plasma cell burden, especially in patients with low percentage of plasma cells on BMA examination. In addition, estimates of plasma cell percentage using CD138 sections demonstrated the highest inter-observer concordance [17]. In our study, CD138 often revealed a higher plasma cell percentage (50% and 39% as assessed microscopically or by CIA analysis, respectively) than that estimated in BMA (29%). As mentioned above, it was generally observed in cases with nodular plasma cell infiltration and marrow fibrosis. One of the possible reasons for this discrepancy is also the fact that myelogram is performed in thin parts of specimen that only contain individual, clearly visible cells, and not in marrow particles where plasma cells are often grouped, and evaluated in BMB. Yet, cytology reports contain information whether there is such a focal aggregation of plasma cells, although this part is not included in the percentage of plasma cells shown in myelogram, so it is unsuitable for statistical comparison and it was left out. The discrepancy has been previously reported on plasma cell assessment in BMB between HE and CD138 stained sections [18]. According to other reports and our experience, CD138 sections should be considered for routine use in the estimation of plasma cell load in bone marrow. Besides, BMB is strongly recommended and adequately examined with immunohistochemistry during follow up of MM, as reported elsewhere [18]. CD138 stained sections allow for excellent assessment of plasma cell counts and distribution in BMB, and can highlight a pattern of distribution indicative of neoplastic infiltration/recurrence in the absence of increased overall bone marrow plasma cell percentage [6,17]. This finding may be of particular importance in the context of post-therapeutic hypocellular marrows where the plasma cell infiltrates may be small and arranged in microaggregates, which may lead to sampling error [6].

The aim of this study was also to highlight the use of CIA, which demonstrated high reproducibility of bone marrow plasma cell quantification [19,20]. This might be of critical importance for the diagnosis, clinical management and prognosis when plasma cell counts low, which makes exact quantification difficult. As shown previously, on computer image analysis, plasma cell quantification was based on color ratios, which reflected the amount of brown CD138 positivity as a fraction of cellular marrow. The system did not actually compute the percentage of labeled cells but rather assessed the relative area of positive stain. With very high levels of plasma cell involvement, the plasma cell area percentage scores obtained by computer tended to be lower than the visual estimates of bone marrow plasma cell percentage using CD138 stained sections. Conversely, with low-level involvement by plasma cells, the plasma cell area percentage scores obtained by computer were higher than those obtained by visual estimation. This suggests that the computer may be more sensitive in detecting plasma cell percentage than visual method. As observed in this analysis, the mean percentage of plasma cells obtained by CIA was higher as compared with BMA, but lower than microscopic analysis in BMB. However, stronger correlation was achieved between the pathologist's and CIA counts than between BMA and CIA.

Numerous prognostic factors have been reported in patients with MM and among them a high plasma cell percentage in bone marrow has also been recognized as a reliable predictor of relapse in cases of treated MM [6]. At the time of diagnosis, approximately 50% of our patients had >50% plasma cell infiltration when analyzed on BMB and nearly the same percentage of MM patients had unfavorable cytologic features, i.e. atypical plasma cell morphology. In 59 patients with follow up data available, survival assessment using Kaplan-Meier analysis revealed favorable prognosis for patients with well differentiated plasma cells and <50% of bone marrow tumor infiltrates estimated in BMB. Similar findings have been reported in the literature [21,22]. These findings support the importance of evaluating BMA and BMB slides in the diagnosis and monitoring the course of MM, as also suggested by other authors [14,23,24].

So, a large part of Scudla et al. report is dedicated to limitations of BMA interpretation, while paying due attention to indications for and specific benefits of BMB assessment [25]. According to the authors, the quantitative and cytomorphological evaluation of bone marrow samples still makes an integral part of the standard algorithm of methods used in MM diagnosis and monitoring, and requires an extremely qualified approach to BM specimen collection, smear preparation and precise evaluation, including their relevance for clinical interpretation [25]. Buss et al. conclude that both bone marrow sections and smears should be examined for suspect MM diagnosis, since neither may be diagnostic alone [26]. Our results are in accordance with these findings. As each analysis has its limitations, combined methods applied whenever possible permit a more thorough approach. At the same time, the presented results give important information on the association between survival and percentage of plasma cells infiltration in BMB, which has not been previously perceived. Accordingly, CD138 immunoperoxidase staining can be recommended for routine assessment of the percentage and kappa and lambda light chains; furthermore, the fact that bone marrow findings correlated with clinical findings may potentially be of predictive value even at the time of establishing the diagnosis of MM. Considering the results obtained, we have to agree with Terpstra et al. that bone marrow biopsy is superior to bone marrow aspirate for direct assessment of tumor load in MM [27]. The possible explanation could be that biopsy offers an insight into the type of infiltration and thus enables a more accurate prediction.

In conclusion, our results showed reduced survival in patients with higher percentage of tumor cells and immature morphology of plasma cells. This result can implicate the necessity of risk-adapted therapy. It would be of great interest to see whether the novel agents such as bortezomib, thalidomide or lenalidomide, as well as their combinations, can overcome the unfavorable impact of these parameters. This issue will be addressed in our future study.

## Disclosure/Conflict of interests

The authors declare that they have no competing interests.

## Authors' contributions

SŠ participated in study design, conceived the study and participated in coordination. EB actively performed part of immunohistochemical analysis. TV was added clinical data. ISB and CŠ participated in study design, regarding cytology section. AN participated in study design and coordination. KL conceived the study and participated in coordination and performed statistical analysis. NJ advised and led the whole group as coordinator. All authors read and approved the final manuscript.
